# Engaging Minority Girls in Organized Youth Sport in Norway: A Case Study of a Project That Worked

**DOI:** 10.3389/fspor.2021.781142

**Published:** 2021-12-16

**Authors:** Siv Gjesdal, Susanna Hedenborg

**Affiliations:** ^1^Institute for Sport and Social Sciences, Norwegian School of Sport Sciences, Oslo, Norway; ^2^Center for Children and Youth Sports, Norwegian School of Sport Sciences, Oslo, Norway; ^3^Department of Sport Sciences, Malmö University, Malmö, Sweden

**Keywords:** self-determination theory, minority girls, sport participation, basic needs, handball

## Abstract

Sport participation is considered a positive pastime endower that can offer a range of positive outcomes for children and youths. It has also increasingly been recognized as a potentially important context for fostering social inclusion for minority youths. Yet across Europe, minority girls are participating in sport to a lesser degree than their majority counterparts. Using self-determination theory (SDT) and the social ecological model as the framework, this study explored the reasons why a particular project aimed at recruiting minority girls to organized team sport succeeded in doing just that. A case study design was adopted to provide an in-depth analysis of how this project satisfied the basic psychological needs of minority girls. Nine girls, four parents, two coaches, and two project team members were interviewed about the project and sport participation in general. Data were analyzed using thematic analysis. Two main themes were identified, a sense of being facilitated and inclusion in the sport environment. The former emphasized the importance of aligning the participation with the girls' cultural norms and values, particularly in the beginning. It also included practical issues such as finances, reminding us that participation in sport is not just a motivational issue. The latter focused on the importance of including the girls in the general sports program, regardless of their athletic abilities at the onset of their participation and creating a mastery environment. Moreover, by removing remediable differences between the minority and majority girls, such as having the right equipment, seemed important to fostering a sense of belonging in the sports club. Additionally, establishing meaningful relationships with coaches and majority counterparts seemed to be a major motivating factor.

## Introduction

While ethnic minority boys in Norway participate in organized sport at the same rate as ethnic majority boys, there is a large participation gap between minority and majority girls, with minority girls half as likely to participate (Strandbu et al., 2019). This gap has also been seen in other western countries, and is problematic since sport participation can offer a range of physical and psychosocial benefits, such as physical fitness, self-esteem, and positive social interactions (Eime et al., 2013; Telford et al., 2016). Moreover, sport is widely considered a potential vehicle for social inclusion of youths with minority backgrounds (Gibbs and Block, 2017; Nobis and El-Kayed, 2019). The Norwegian government, for example, points to organized sport as one aspect of the community that can, and therefore should, play an important part in establishing contact between majority and minority populations (Norwegian Ministry of Justice and Public Security, 2016).

Considering the rather dire participation rates for minority girls, examining how we can engage them in organized sport seems warranted. Although the topic of minorities and sport has received a surge in popularity recently, many studies examine only structural or sociocultural aspects related to barriers, focusing less on the experiential dimensions of sport participation (Spaaij et al., 2019). Yet, previous research has found that minority girls' decision to engage or not engage in sports is a result of both macro-level factors, such as culture and the local community, as well as more personal factors located in the microsystem (Thul and LaVoi, 2011), similarly to their majority counterparts (Craike et al., 2009). It is therefore important to not negate the influence of intra- and inter-personal aspects when attempting to understand how to engage this subgroup in organized sports. For example, Zhang and Solmon (2013) called for an integration of self-determination theory (SDT) and the social-ecological model as a theoretical basis for understanding physical activity behavior. The social-ecological model is a comprehensive framework, concerned with the interrelations among diverse environmental conditions and individual factors in human behavior and health (Stokols, 1996; Bronfenbrenner, 1999). Whereas, the social-ecological framework typically lacks the specificity to identify influential determinants of behavior, Zhang and Solomon argued that SDT alone fails to consider the potential influence of a broader specter of structural factors. Thus, combining SDT with ecological reasoning will allow for valuable research on how both the structural context and more person factors may contribute to sport participation.

Ryan and Deci (2007) argued that because sporting activity demands exertion, energy, and focus, it epitomizes motivation; people being moved to act. Furthermore, motivation is thought to be closely related to the quality of sport participation (Vallerand, 1997; Ryan and Deci, 2007; Roberts, 2012). This makes motivation a particularly relevant factor to study when trying to understand engagement in sport. In the present study, in line with Zhang and Solmon (2013), we drew upon SDT (Ryan and Deci, 2017) to study this topic. According to SDT, the satisfaction of three basic psychological needs is essential to the entry into, and the continuation of, sport participation (Ryan and Deci, 2007, 2017; Curran et al., 2016). It therefore follows that to engage minority girls in organized sport, one must satisfy the participants' basic psychological needs, namely the need for autonomy, competence and relatedness (Ryan and Deci, 2017; Vansteenkiste et al., 2020). Autonomy refers to self-regulated behavior and experiences associated with a sense of volition and congruence, competence is reflected in a feeling of effectance and mastery, whilst relatedness is defined as feeling integral to social structures outside oneself, akin to belonging, and connectedness.

Previous research has associated minority girls' sport participation with the basic psychological needs (Farello et al., 2019). Our aim was therefore not to examine whether the basic needs were important to minority girls' decision to participate in sports, but rather how these needs can be satisfied. According to SDT, supportive socializing agents account for personal and situational characteristics that influence participants' need-satisfying experiences (Vansteenkiste et al., 2020). It therefore follows that most studies investigating basic psychological needs focus on the influence of the psychosocial environment, and mainly the coach. Findings overwhelmingly suggest that by being supportive, coaches can satisfy all three needs (Mageau and Vallerand, 2003; Thul and LaVoi, 2011; Adie et al., 2012; Curran et al., 2016). However, researchers have been calling for more studies attempting to understand the distinct needs of female youth members of minority populations, in order to help them reap the potential benefits of sport participation (Middleton et al., 2020). In fact, the theory of basic psychological needs does not assume a one-size-fits-all perspective, and research on specific subgroups is important because how a particular need is satisfied and the salience of that need may vary as a function of, for example, cultural background (Ryan and Deci, 2000, 2017). For instance, it has been suggested that individuals from non-western societies may be more open to influence from their parents, being autonomous in their acceptance of parental decision-making on their behalf (Ryan and Deci, 2017). Thus, in order to instruct socializing agents on how they can support the needs of minority girls in the sport context, we need to know more about how basic need satisfaction manifests itself for this subgroup within that context, and not just rely on research conducted on majority samples.

It seems evident that coaches and parents can be influential in terms of participation. However, circling back to the critique by Zhang and Solmon (2013), although SDT is valuable in understanding intra- and inter-personal aspects related to sport participation, it may be somewhat limited in answering the question of how to minimize the participation gap between minority and majority girls. In fact, a recent report on the subject strongly encouraged the consideration of the complex ecological factors that influence sport participation (LaVoi et al., 2018). As aforementioned, the more structural and socio-cultural influences on sport participation in refugee and immigrant populations has received some attention. For example, transportation constraints, lack of funding, little government policy and investment, lack of inclusive sporting practice, and unsupportive community norms have been found to constrain sport participation in minority girls (LaVoi et al., 2018; Spaaij et al., 2019). However, this research has focused mainly on barriers, with little focus on how these aspects can facilitate participation. Moreover, according to social ecological reasoning, it is important to acknowledge that social and personal variables are occurring within distinct environmental settings, and investigating them specifically as it relates to the structure of these contexts have previously been emphasized (Bengoechea, 2002; Sallis et al., 2008). Unfortunately, to our knowledge the only study to look at more macro-level factors and basic psychological need satisfaction in minority girls' decision to participate in organized sport was the study by Farello et al. (2019). Specifically, this study showed that a lack of community support was related to their need for relatedness. Although these are interesting findings, research from the US is not necessarily applicable to Norway. While sport in the US is closely related to the school domain, there has been little or no cooperation between the educational system and the voluntary based sport clubs that largely offer sporting activities in Norway (Støckel et al., 2010). Moreover, the study by Farello et al. looked at general experiences with sport and physical activity, and we would argue that it is crucial to separate the experiences of organized sport from other types of physical activity, and that continuing this line of theory driven research in different context and countries is crucial to offering opportunity for contextual comparisons and contrasts.

Accordingly, the aim on the present study was add to our understanding of sport participation for minority girls. By combing SDT with an ecological perspective, we set out to examine how a particular project succeeded at engaging minority girls in organized sport, by focusing on the girls' basic psychological needs but also the broader context surrounding the sports program. Specially, we conducted a case study of a sport club-based project, The Colorful Sport Project (CSP), because of its success at engaging minority girls in organized sport. It was hoped that the findings from the study can contribute to our knowledgebase on minority girls' participation in organized sport.

## Methods

### Study Design and The Case

A single explanatory case study design was employed to gain in-depth knowledge of minority girls' engagement in organized sport, as this method is particularly apt at addressing “how” and “why” questions relating to minority girls' sport participation (Yin, 2018). The engagement of minority girls was the critical case we wanted to study, and the CSP was chosen as the context to study this case because of its success. Specifically, the aim of the CSP was that minority girls should represent 11% of the participants, reflecting the demographics of the local community. Amazingly, at the end of the project this subgroup accounted for 21.3% of the participants (Vadsø Turnforening, 2014). Moreover, these numbers seemed to hold steady several years afterwards. The project was organized by a local sport club in cooperation with local authorities responsible for settling and supporting newly arrived asylum seekers and refugees. The club is located in a small town in the scarcely populated northern part of Norway and offered handball and gymnastics in their regular sport program. The project itself ran over 3 years, that is three athletic seasons, and consisted of four distinct parts; (1) creating a minority only group where girls 13 years and older could try different sports, (2) offering open sport hall Sunday sessions for families with no requirement of club membership, (3) establishing an after school-program for first and second graders centered around sport, and (4) actively recruiting minority girls to established teams within the club.

The CSP provided the analytical frame for interpretations, whilst the units of analysis were important stakeholders, namely minority girls, their parents, coaches as well as other project members. The sources of data were mainly interviews, yet we also relied on funding applications, project rapports, and member statistics to give background and context. Collecting data from not just the girls, but also parents and other adults involved in the project allowed us to view the phenomena through different lenses, furthering our understanding (Baxter and Jack, 2008). Interviews with girls who had been or were currently engaged in sport as a result of the CSP project served as the main source of data, while interviews with coaches, members of the project management, and parents served to expand and nuance the information given to us by the girls.

The participants in the present study were nine girls between the ages of nine and 19 and four parents, all settled refugees or asylum seekers from Nigeria, Eritrea, Ethiopia, and Afghanistan. The participants had all been in Norway for 3 years or longer. We referred to them as newly established minorities in the present paper, as some refugees and asylum seekers wish to be publicly acknowledged as minorities once they have been in the country for some time (Spaaij et al., 2019). We also interviewed the CSP project leader and a team member who both served as club coaches, plus two other club coaches. The girls were recruited through the project leader, and were currently or previously involved in organized handball in the club and had started their participation through the CSP project. Most of the older girls had participated in the minority only group, the girls 14–16 years had been actively recruited to the club, while the younger girls became involved with the club through the open hall sessions and the after school-program.

### Data Collection

Formal approval for the present study was obtained from the institutional review board at the Norwegian School of Sport Sciences, as well as the Norwegian Center for Research Data. The participants were recruited through the CSP project leader. The organizers/coaches, parents and girls above the age of 15 years consented themselves, while consent was obtained from parents for girls under 15, who were informed that they could decline participation even if their parents had consented. Participation was voluntary, and consent could be withdrawn at any point. However, several researchers have questioned the use of such formal processes of consent with populations similar to the one in the present study (Gibbs and Block, 2017; Spaaij et al., 2019). We therefore also verbally informed participants about the nature of the study and how they could help, and made sure to ask them at the end for their input on how one might improve minority engagement in sport settings. Finally, in order to maintain anonymity, age, and ethnicity will not be referred to when individuals are quoted.

The interviews were semi-structured carried out by the primary researcher. The interview guide was developed following an extensive review of the literature on minority girls' participation in organized sport as well as SDT, and the primary researcher's understanding of the organized team sport context in Norway. Specifically, the primary researcher has several years of experience as a youth sport coach in Norway and has a thorough understanding of this context. The result was a series of open-ended questions, not related specifically to SDT constructs, but rather general questions about their experiences with the sport and/or the project. The use of open-ended questions allowed the participants to share their experiences, and to reflect on what was important to them. We were cognizant that we were interviewing children of different ages so we made sure to include more direct questions if the open-ended questions appeared difficult for them (Irwin and Johnson, 2005). This did seem to be the case with the youngest girls, resulting in some shorter interviews.

To lessen the burden on participants, interviews were held at a place of convenience for them at a time of their choosing. The interviews lasted between 16 and 86 min, and were audio recorded and transcribed. Key questions posed to the girls focused on why they decided to try handball, why they continued with it, and their experience with participation (e.g., “what is the best part about handball?”). Key questions presented to the parents were similar, centering on their view of their daughters' sport participation (“how did your daughter become involved in handball?”). Also, aspects related to the larger context beyond the intra- and inter-personal was probed when brought up by the interviewees. As parents and children were both interviewed about the same topic, a conflict of interest may have been present. For example, some girls may have wanted to avoid certain topics, such as religion, due to loyalty toward their parents. To reduce some potential pressure on the girls, we did not ask them directly about the role of their parents, although we did probe the topic further if they themselves brought it up. Key questions posed to organizers and coaches focused on the background for, implementation of, as well as the outcomes of the project. During these interviews it was natural for us to also pose questions regarding the larger context surrounding the local sports club (e.g., “how did local community respond to this project?”).

### Analytical Approach

The data were analyzed using a reflexive thematic analysis. This entails identifying and retrieving themes form the data, produced at the intersection of the researcher's theoretical assumptions, analytical resources, and the data itself (Braun and Clarke, 2019, 2020). We used a primarily inductive approach, in which codes and themes were developed from our interpretation of the data content, allowing us to remain reflexive in relation to themes that did not fit with our theoretical framework (Nowell et al., 2017). This means that we did not create themes based on SDT, but rather created more broad themes and then discussed these in relation to SDT in our discussion section. Furthermore, we recognize that there is always an analytical risk present when interpreting what is said in interviews, perhaps even more so with children. We were therefore cautious not to interpret answers as uncontestable truths but rather as indicators of their subjective experiences.

Computer-assisted qualitative data analysis software (MAXQDA plus v18.2.0) was employed to aid the analysis. We based ourselves on Braun and Clarke's (2019) six-stage process because of its flexibility and potential for delivering rich and complex understandings. Familiarization was the first step, in which the data were read and reread several times. In this step, some general ideas about what was in the data was noted. Second, initial codes were generated based on key words and concepts, focused both on the semantic content of what was said, but also the more latent meanings in the data. This resulted in rather broad codes such as “friends” and “costs.” Third, the initial codes were then sorted into potential themes based on broad patterns of meaning across the entire data set. This resulted in a collection of candidate themes and sub-themes, such as “gaining new friends.” Fourth, these potential themes were refined and then compared to the entire data set to assess whether they fit the data set. In step five the themes were defined and given names. Last, in step six, the results section was written to offer the reader an understanding of each theme.

In order to increase the trustworthiness of the data collection and analysis processes, several measures were taken (Nowell et al., 2017). First, the researchers were familiar with the theoretical background for the study, as well as the case study topic and context. Moreover, all the data collected was attended to, contributing to our interpretations. For example, the documents were given attention both before and after interviews, in order to both generate questions and to offer supplemental information (Bowen, 2009). Furthermore, some of the interviews with the girls and their parents were conducted before interviews with other stakeholders, and some after. This allowed us to use the information given to us by the girls to guide our interview with stakeholders, and vice versa. Finally, throughout this data collection and analysis the primary researcher regularly discussed the topic with the second author.

## Findings

Two main themes were identified. The first, a sense of being facilitated, concerned experiencing the sport participation tailored to these girls specifically. It reflected both practical elements such as financial issues and information, but also more personal ones related to values and norms. We refer to this as “a sense of being” because what appeared important is the girls', and their parents', perception of these aspects. For example, it is not information in and of itself, but rather how the information is perceived. The second, being a part of the sporting environment, was more reflective of the experience they had in organized sport, and the importance of being included both socially and in terms of the actual sporting activity.

### A Sense of Being Facilitated: Information

In order to recruit minority girls to the sporting program, a first step was informing them and their parents of what the club had to offer. It seemed that much of the information centered around organized youth sport in Norway, both various practicalities and the benefits of doing sport. However, the main point did not seem to be content but rather how they disseminated the information. This had drastically changed from the beginning of the project, from formal to more personal. For example, sending information sheets to nurseries and schools had not yielded results. Both coaches, project members, and parents talked about how important it was to have a personal touch when informing people, and therefore searching for forums where parents could be contacted in person, such as the church or the reception center, was vital. As a coach and a member of the project explained:

*...you must have someone that's is on, like [project leader and project member]. That are very committed and are trustworthy people…And willingly go, like they, go visit them and talk to them*. (Coach)

*It seemed as if it was very important to the parents that I came home to them and talked to them, so they knew that ok when I send my kid to training she's there, I can talk to her if there's anythin*g. (Project member)

The parents themselves specifically talked about how much they appreciated that members of the club came directly to them, even visited them, when there was information they needed. There was also a concerted effort to disseminate information in various languages, such as Somali, Arabic, and Farsi, and its importance was emphasized by a member of the project: “*But I am sure it helped, if just to, just to give them the right information, so I think they felt that hey we're welcome here. They want us to join, they have actually translated this*.”

### A Sense of Being Facilitated: Practicalities

The aspect of practicalities was related to the broader context surrounding the local sports club. For example, all of the adults and some of the older girls interviewed mentioned the importance of financial costs to the girls' opportunity to participate in sport. Members of the project management team admitted that costs ended up being a more critical factor than they had anticipated. This was corroborated by a father who explained that receiving help from the club in seeking financial support from the municipality made a great difference:

*They [mentions members of the project management] help us with a lot. Clothes, and the invoice. They helped us with NAV [Norwegian Labor and Welfare Administration]. We got the invoice [from the club] and they helped us get it to NAV. They paid it, and that's good. Because we do not have jobs. When we get jobs, we will pay*. (Father)

An interesting detail was how the girls themselves focused far less on the cost issue compared to the adults, with the younger girls not mentioning it at all. Moreover, another practicality that the adults focused more on was the local environment. Specifically, a small municipality seemed to ease the inclusion of these girls in the local club, as the families were not dependent on transportation. In addition to easing participation, living in a small community also offered a feeling of safety as one mother explained: “*The children go to training alone…you don't worry when they're there, it's all so close to us*.”

### A Sense of Being Facilitated: Values and Norms

Both the girls and the parents were adamant that it was the girls themselves who initiated participation in organized sport, illustrated by one mother saying: “*And if the children want to do sport, it is them who opens that door first…they decide themselves, to start, and what sport*.” Indeed, it did not seem as if any of the girls experienced any encouragement or pressure from their parents to start their participation in sport. However, the desire to participate often came after the sport program had been adjusted to accommodate both the girls' and the parents' values, indicating the importance of accommodations made on the structural level. Initially many of the parents had been skeptical. According to the project manager, it was only when the minority parents had questions that they started actively seeking out the parents, and the girls really started showing up:

*That communication was not planned, not at all. But we saw that, it works. So, we invited some [parents], after we saw that there are some key people in the different cultures, the different communities. So, we invited four mothers, that all had two or up to five girls…so we invited them to a meeting with us in the project team. To talk about girls and sport, finances and asked about what they wanted to know. We saw that the most important thing was that they saw our faces, got to know us, trusted us*. (Project leader)

Even when the girls started to show up at the sport hall, there was still a bit of facilitation in order to keep them coming back. For example:

*We had hijabs made, like sport hijabs that were meant to be part of the kit. And that, although they are not used a lot, I think it helped. Because, I think parents, and the girls themselves, got the impression that hey we are trying to facilitate you. We do not mind you using hijabs but use this one because it doesn't have needles and decorations*. (Project leader)

*What we had to focus a little extra on, when they started playing games and such, was the aspect of physical contact. So, I think, we had to practice being close to each other and touch each other and hold each other*. (Project member)

It seemed that this facilitation was most important in the beginning, after a while it did not seem that crucial anymore:

*In six months, we had girls who in the beginning couldn't go into the sport hall because there were boys there, and had to wait outside until all had left, to after six months being able to play against an all-boys team in front of many spectators in the sport hall. It took six months right. They felt safe, the parents knew what, where, we made sure of that*. (Project member)

When deciding to try out sport the girls seemed concerned with some extrinsic values associated with the participation. The most mentioned by the girls was learning the language, and one of the girls mentioned this as her main motivation for initially participating: “*Because I couldn't speak Norwegian, and those who were in my class, the girls said I could learn Norwegian if I started handball. And so, I started handball*.” This sentiment was supported by the parents interviewed, who agreed that organized sport had been important for their children's language development. Moreover, the parents highlighted the importance of activity for physical health, illustrated by a father: “*Because children need to go out. Exercise, football, handball. Go for a walk. Children need, all people need that*.” One of the girls corroborated this, stating that her mum encouraged her continued participation in sport mainly because of the health benefits:

*She has motivated me like, to do this. Even though she sees that I have a lot of homework right. That it's good for my brain, that you should have, take some breaks. I know it's hard with school and stuff, I want you to learn, but exercise is important too, for your health*. (Girl)

A competing value that seemed to interfere with sport participation was the importance placed on education. One coach commented on how career-focused the minority girls were: “*School is very important. While some of the others [ethnic Norwegian girls] can be a bit more relaxed*.” Some of the older girls had discontinued their participation when they turned 16: “*There was a lot to do at school, lots of homework. So, then I didn't have more time for it [sport]*.” The younger girls presented similar attitudes, in that at some point in the future they expected to have to choose and would then choose school.

### Inclusion in the Social Environment: Developing Meaningful Relationships

All the girls mentioned aspects related to social relationships as important, and being with friends seemed to be a major factor in the girls' decision to join the club. An informal word-of-mouth marketing was pivotal in making them want to try handball, as one girl commented, “*Because one of my friends, she used to brag about how nice it is. And so, I wanted to start, so I started. And I thought it was really good*.”

All the girls mentioned gaining new friends through their participation, and believed it benefitted them beyond the sporting context:

*Before I spent my time watching movies and stuff. But most of my time I now spend on handball. That and the social stuff, getting to know people...Many of them I wouldn't have gotten to know otherwise. When I spend more time with people, I get to know them more. And it's easier for you to work with them at school, and other places, anywhere really*. (Girl)

This was corroborated by the parents, who underlined the importance of being social, with one mother saying “*It's boring to be home. Not good to be home alone, they don't learn, they don't get to know people*.” Many of the minority girls made friends that were ethnic Norwegians. One of the coaches, who worked as a teacher at the local high school, commented on the positive effects of this, stating: “*We see that when they come here* [high school]*, they know each other. They are much more comfortable in their interaction*.” However, in this regard there seemed to be a difference between the girls who participated in the normal club activity and those who only participated in the group for minority girls only. As one of the girls who had participated in both commented: “*You can see a difference between me and them* [the minority girls who participated in the minority group only]*, that I have more contact with the other Norwegian girls than they do.”*

A good relationship with the coaches also seemed imperative for several of the girls, with one noting that the coaches were the ones who had given them the best reception when they first joined the club. According to the project management a close relationship with the adults at the club was almost a symbol of status for the girls:

*They are very eager to give us a hug right. And when people ask how do you know her, they say well she's friends with my mum. And I see that they are very like, my mum has Norwegian friends. That it maybe means something to them. It sounds weird to say, to promote myself like that, but still I see it. And I might have had a special care for the minority girls*. (Project leader)

It also seemed that gaining a sense of belonging within into the club and the local community was experienced as valuable. When asked how it felt to be a part of the handball club, one of the girls answered:

*That I'm worth as much as the others. That there's no difference between me and them, even though I use a hijab. I use long clothes, but there's no difference. I am a girl, they are girls, I can play handball…. You can say that I don't become my culture*. (Girl)

The project had received political support, and the participants got a lot of media attention. One of the members of the project talked about how the minority only group perhaps had not provided the girls with social inclusion directly, yet the experience appeared to have given them a sense of pride and served to inspire others:

*It's given them a lot, I think they have felt that, this is cool, we're cool. And the younger girls have been there and seen this, and seen that, this is good, she's 20 years old and Muslim and a girl, and she plays handball*. (Project member)

Developing social relationships was not limited to the girls. The parents explained that they had gotten to know people in the community through the club. There had been a concerted effort to get parents of minority girls to volunteer. A member of the project team explained that many parents had understood that their daughter's sport participation could mean something for them as well: “*Many have discovered that this is actually a great opportunity to get to know more people, and networking, how important that is*.”

### Inclusion in the Social Environment: Being a Part of the Sports Team

Being a legitimate part of the sports team seemed important to the girls. Specifically, this meant participating as a part of the normal sport program within the club. This was somewhat of a challenge for the club because the minority girls generally started later than their majority counterparts and were therefore naturally behind in terms of skills. However, this seemed to be helped by a sport program with a clear mastery philosophy, as one coach commented:

...*the focus is player development. And that they get playing time. Right, you don't travel far to play 5 minutes, and sit the rest of the time on the bench. That's boring, then they quit right away*. (Coach)

That this was the focus within the club was supported by the girls, with one stating that:

*They [coaches] don't care a lot about, when we're playing a game, the scoreboard. They care about us trying things, mastering things. Because it's not now that we have perform and such, that's like when we're sixteen. They care mostly about us learning*. (Girl)

In line with this mastery focus, many of the girls mentioned cooperation as something they enjoyed with their participation in handball, with one girl stating “*I like that you get to play with others on the team, you can talk to them about what to do, for example a set play, or find a way to work together*.” There appeared to be a clear focus on helping each other out and working together, according to another: “*I like that we work together, because I have a lot of friends there, and they help me and give me tips on how to play*.” This was supported by one of the coaches:

*The youths are very good nowadays. They are so inclusive. Like say, ten, twelve years ago, if someone couldn't catch, or couldn't throw, they would roll their eyes. They don't do that anymore. Now they go over and tell them how to do it, help… I think that's very important*. (Coach)

Learning and mastering skills seemed to be a big part of what made the participation in sports enjoyable for the girls, yet there were some challenges getting the older minority girls to understand the value of development and that it takes time to master new skills:

*You will meet challenges regardless, but its extra difficult for those who are older. They take it so seriously, they take it hard. A seven-year-old will forget it by the next practice, be disappointed but then it's over. But these girls feel like a failure*. (Project member)

The importance of the mastery philosophy was perhaps most evident when welcoming new girls to the club. They were included in the team straight away, during both practice and matches. Although they received extra help when needed, they still partook in the same type of drills that the rest of the team worked with. The importance of this philosophy was best illustrated by what happened with two girls who tried to join a team that did not share this philosophy:

*I had two girls with me, both coming from the reception center…who wanted to start playing handball.…but what happened was that they were left to themselves. So the one girl told me after a few practices that no I don't want to. We just stand behind the goal throwing the ball, me and the other new girl…There was a large difference in ability level, and the coaches were so focused on the level, we are going to reach high. And to then start building someone up from the ground, they weren't interested in that…that team still does not have a single minority girl, and haven't had one since they started*. (Project member)

When asked what the best part of practices were, the girls were unison in that being game time. Although this may at first glance seem counter to the importance of mastery, the girls were more focused on the notion of play rather than normative competition. Moreover, partaking in tournaments and away games were an essential part of the handball experience. This was exemplified by one girl being asked about a good experience she had had with handball, replying “*The first time we went to another town to play matches*.” A coach stressed the importance of matches: “*They learn a lot when they're are there, handball. There are so many games. They see what they have to do. I as a coach can't show them that during practice. It is not the same*.” The importance of partaking in these away games was also related to the social aspect of participation. Because of the large distances of the northern part of Norway, tournaments and away games require a lot of traveling and often spending the night. This seemed to make competition very enjoyable, with one coach noting that: “*It is where you create the connection between the girls, and the parents that come with*.”

In addition to a mastery focus, an important tool for ensuring that the girls felt a part of the team was the club kit, bought and given to the girls free of charge:

*When we bought the kits for them, that was exciting in itself. They came with us to the shop, tried them on, got their names printed on…they even used them at school. It wasn't just at practice. It was so important for them to show that hey I am also a part of this*. (Project member)

This sentiment was supported by the girls, showing that the kit was crucial to the feeling of belonging:

*...I wore every day the same, pants and t-shirt let's say, and then [her coach] said, when I started playing handball, come with me, and we went to the sporting goods store, what sport clothes that the others have do you want to buy, like pants and a sweater and stuff. Then she bought that for me, even socks. Shoes and everything. I like felt that I became a part of it then…I felt that it was nice of her, because we couldn't afford that, right*. (Girl)

## Discussion

Given the dire participation rates in organized sport for minority girls in Norway (Strandbu et al., 2019), this study focused on understanding how we can satisfy minority girls' basic psychological needs through a local sport-club based project. The results, first of all, show the importance of access to the success of the CSP. Therein, the significance of the financial cost of sport should not be ignored, especially seen as a recent report shows how the cost of organized sport in Norway has increased just from 2015 to 2021 (Jacobsen et al., 2021). What was particularly noteworthy, however, was how some girls explained that once they received some form of financial help, they felt equal and included. This can be referred to as equity, defined by the World Health Organization as the absence of remediable differences between groups of people (WHO, 2020). Specifically, in situations where there are differences between groups that create barriers for sport participation, such as finances, it is only when these differences are remedied that we can talk about equal opportunities for sport participation. This represents an example of how aspects beyond the intra- and inter-personal level can influence a sense of relatedness, essentially showing that basic need satisfaction is not purely a result of the microsystem.

Related to access, it is important to note that the CSP was located in a small town in northern Norway. This meant few competing extracurricular activities, very safe neighborhoods and little need for transportation, similar to the more structural elements found in previous research (Spaaij et al., 2019). Although the girls did not focus greatly on the issue of location, its potential contribution to the success of the project should not be ignored, particularly since it was mentioned by several parents. Moreover, a recent report found that sports clubs in smaller and more rural municipalities in Norway are better at recruiting families from lower socio-economic backgrounds, tying this to the importance of costs aforementioned. Thus, future work is needed to assess whether a similar project can be equally successful in a larger city.

Although access appeared important, it seemed like a necessary but not sufficient factor, and beyond this, several aspects linked to the basic psychological needs were found. Autonomy, for example, was not mentioned overtly by the girls yet probing the data yielded a different story. Indeed, fostering the girls' sense of autonomy seemed to be done by aligning participation with their values and norms, actively communicating with the girls and their parents coupled with a considerable willingness to tailor activities to suit the girls. This corroborates previous findings underlining the importance of talking to the girls themselves when including them in sport (Lowrey and Kay, 2005; Thul and LaVoi, 2011). This can be seen as autonomy support, essentially valuing the importance of personal relevance (Mageau and Vallerand, 2003). Specifically related to the present findings, this autonomy support was characterized by cultural sensitivity, for example in terms of uniform requirements (Olliff, 2008; Thul and LaVoi, 2011). This corroborates previous research finding that flexible dress requirements are important for minority women's participation in sports (Cortis, 2009).

Also corroborating earlier research (Langøien et al., 2017; Spaaij et al., 2019), is how the local political environment seemed to have an impact on sport participation through the readiness to make the aforementioned adaptations to meet minority girls' needs. Moreover, the attention given, both from the media and the local community itself, seemed to create a sense of pride for the girls themselves. These findings represent interesting links between the macro-level and autonomy and relatedness need satisfaction. Although the link to relatedness has been reported previously (Farello et al., 2019), the association to autonomy is a novel finding that further underlines the importance of considering the local environment when working on increasing organized sport participation in minority girls.

An interesting finding was that it seemed as if aligning the participation with their values and norms was most crucial at entry into the sports program, and that this manifested itself somewhat differently once the girls were an integral part of the context. This moves beyond previous research underlining the importance of gendered spaces and allowing culturally appropriate clothing (Thul and LaVoi, 2011), by showing that although this was also important herein, its relevance seemed to decline over time. In fact, after only six months the girls felt comfortable playing against boys, in handball, which requires bodily contact. As such, it could be that entry into sport program is the most sensitive phase, yet if one succeeds in meeting their personal values and norms immediately the special accommodations may not be necessary over time. However, this requires more research moving forward.

Related to personal relevance was the importance of informing the girls and their parents about organized youth sport in Norway, and its benefits. According to Langøien et al. (2017), one of the main reasons why minority populations in Europe were not as active as the majority was a lack of knowledge of physical activity and health. The CSP focused on the extrinsic value of sport, for example, learning the language and physical activity, both of which have been found to be reasons for sport participation in similar samples previously (Olliff, 2008). Ryan and Deci (2007) argued that not all parts of sport participation will be intrinsically rewarding, and that extrinsic values can be fundamental for sustained engagement. Thus, a focus on potential benefits of sport participation may be a fruitful avenue for promoting participation, of course in combination with a program that also promotes intrinsic interest.

Both the girls and the parents were adamant that the girls themselves initiated participation, free from any pressure or probing from parents. This contradicts research done on mainly majority youths in Norway which has found that parents are an important initiator and motivator for sport participation (Stefansen et al., 2018). On closer examination of the data, it did seem as if a good deal of the effort regarding recruitment and facilitation had been aimed at parents, suggesting that parents were at least part of the decision to partake in sport. This is not to say that the girls were not autonomous, as there can be cultural differences in where the line is drawn for personal boundaries and acceptance of influence from others. In such instances, autonomy can be experienced by accepting and internalizing choices made by trusted people, such as parents (Ryan and Deci, 2017; Vansteenkiste et al., 2020). This is also in accordance with previous research from the US, showing parents need to approve of physical activity endowers for it to be a feasible pastime for minority girls (Thul and LaVoi, 2011). Understanding these types of nuances are imperative for us to ensure basic psychological need satisfaction in youths from various cultural backgrounds.

An example of the mesosystem, that is interactions between different part of the microsystem, was how the importance placed on education acted as a barrier for sport participation. This is perhaps not surprising as previous research in Norway and the US has shown that immigrant parents generally expect girls to spend a great deal of time studying (Walseth, 2006; Thul and LaVoi, 2011). Yet this is also an area where the Norwegian context differs from the US, in that there traditionally has been little or no cooperation between the educational system and the youth sport domain (Støckel et al., 2010). It may be that Norwegian sports clubs could benefit from a closer connection with the local schools, or at least focus on creating a program that combines well with the academic school year.

Once the girls had access to sport participation, their motivation for starting and continuing their participation seemed to align well with the other two basic psychological needs. Relatedness seemed to be most apparent, as being with friends, making new friends, and feeling a sense of belonging was a major part of both why they wanted to try sport, and why they continued their participation. The importance of peers for young peoples' commitment to sport has been well-established previously (Ullrich-French and Smith, 2009; Farello et al., 2019), here with the added layer of creating social relationships with majority counterparts. The importance of friends in recruiting minority girls may be particularly relevant due to the lack of parental initiation found in the present study, in contrast to that found in research with a mainly majority sample (Stefansen et al., 2018).

A less overt example of relatedness was found, namely the importance of a personal touch when communicating with the families. The members of the project actively sought out parents, visited them and stayed in contact even once participation had been established. This seemed to create a sense of community, and developed trust between parents and the club. According to Vansteenkiste et al. (2020), experiencing a strong bond to a socializing agent can provide a starting point for internalizing the values of an activity. In this instance, it could be that this bond allowed the families to start internalizing the value of sport, based on what was being communicated by the project members. This supports the use of community liaisons to actively link the youths and their families to sport clubs, as advocated in previous research (Gibbs and Block, 2017). This is obviously a costly measure, yet one that might be worth it if one is serious about engaging this subgroup in organized sport.

An interesting and unique finding was how the inclusion of the parents in volunteer work for the club lead them to make social connections with others and become more visible in the local community. This essential shows how organized sport participation for the children can lead to a sense of relatedness for the parents as well, if one succeeds in including them within the club. This finding may represent a specific characteristic of the Norwegian context. This type of voluntary work is referred to as *dugnad*, a cultural praxis based on the traditions of cooperation, where the work is done as collective (Simon and Mobekk, 2019). Indeed, in Norway, youth sports are essential built on the notion of *dugnad*, and it is almost expected that one contributes as a parent (Støckel et al., 2010; Folkestad et al., 2017). Although this may act exclusionary, the CSP actively worked on including the parents in this praxis, essentially making it a positive feature instead.

The meaning that the girls attached to being a part of the sporting environment was also centered around sport-specific aspects. It seemed important for them to be a part of the team, and to feel a sense of mastery. This can be linked to the need for competence, which has been associated with sport commitment previously (Ullrich-French and Smith, 2009). The project seemed to facilitate this by ensuring a mastery climate was created by the coaches. This may be particularly important for minority populations as they tend to start their participation later and have less experience with sport, which can lead to feelings of physical incompetence (Farello et al., 2019). Indeed, Cortis (2009) found that a lack of skills, and general self-consciousness, could act as a barrier for sport participation for this subgroup, further underlining the importance of focusing on mastery rather than normative performance.

The coaches described how they used differentiation, a pedagogical tool closely related to the aim of inclusion as it values everyone's achievements equally, and promotes the idea that all participants should be able to succeed regardless of ability level or personal circumstances (Morgan, 2017). This is in accordance with previous recommendations for creating inclusive sport programs by limiting the negative aspects of competition and instead focusing on learning and development (Olliff, 2008). The importance of this approach became apparent in the present study as the one team that operated with a more performance-oriented climate failed at recruiting minority girls. This is unsurprising, because the very nature of a performance-focused environment is built on the value of normative competition, with little room for diversity or mastery experiences for a heterogeneous group (Nicholls, 1989). Indeed, recent research showed that a mastery climate created by the coach, characterized by little intra-team competition, lead minority athletes to experience a greater sense of inclusion within the team (Van Yperen et al., 2021). Moreover, this focus on mastery could also have been important for the satisfaction of relatedness through friendship, as mentioned earlier, as previous research has found that a mastery climate is related to positive peer relations in youth sport (Ommundsen et al., 2005).

## Strengths and Limitations

We believe that the present study has several strengths. First and foremost, it contributes to our knowledge base on how to engage minority girls in organized sport. This is an important topic that will remain relevant in the years to come. Second, by collecting data from several sources we gained a more nuanced picture of the project and its influence on the girls' sporting participation than we would have if we had just collected data from a single stakeholder. Last, in contrast to a lot of the sport psychology research conducted on this subgroup, choosing to have a theoretical backdrop for our analyses means that our findings are more easily compared to previous and future research (Hatzigeorgiadis et al., 2013).

The present study is not without its limitations. First, only including girls who joined the sport club may present some biases in our data. Future research should aim to reach girls who chose not to participate in order to learn their reasons. Second, we decided not to differentiate between the minority girls based on their specific ethnic background. We would like to acknowledge that there may of course be differences based on where they fled from, however, in this project their similarities likely outweigh their differences. Third, the age range of the girls is rather large. Future research would benefit from differentiating based on age. Last, some of the findings could be also appear in similar studies with majority girls, for example the notion of costs and the importance of friends. We do not want to assume that any of these aspects are definitely tied to their status as settled refugees and would therefore advocate for intersectionality moving forward (Spaaij et al., 2019). This can also be related to potential gender differences, which we did not study. We agree with Middleton, Petersen (Middleton et al., 2020), that this is a line of inquiry that deserves attention.

## Conclusions

The present findings remind us that although we may believe motivation is of the utmost importance when investigating sport participation, its importance assumes access. Without access, you can have the best quality motivation possible yet never have the opportunity to engage. This can perhaps be a blind spot for sport psychology researchers, in that we, due to our privilege, seldom must acknowledge that lack of access is an issue, even in western societies. A lack of access to sport can produce and reproduce social inequalities (Nobis and El-Kayed, 2019), and cannot be solved as a purely motivational issue. This underlines the value of combining the study of motivation with the social ecological model, to offer a more comprehensive picture of sport participation, or a lack thereof. Specifically, findings show that gaining access to the sport itself relied on a combination of more structural facilitators, such as no transportation needs, financial support and a local community willing to make adaptions to the sport program, as well as more motivational elements, such as tailoring the sports program to the specific needs of the girls and including both them and their families in the club.

## Data Availability Statement

The raw data supporting the conclusions of this article will be made available by the authors, without undue reservation.

## Ethics Statement

The studies involving human participants were reviewed and approved by Norwegian Center for Research Data. Written informed consent to participate in this study was provided by the participants' legal guardian/next of kin.

## Author Contributions

SG has contributed to the development, implementation, interpretation of this study, and also written the manuscript. SH has contributed to the development and interpretation of this study, and has given commentary on the manuscript. All authors contributed to the article and approved the submitted version.

## Conflict of Interest

The authors declare that the research was conducted in the absence of any commercial or financial relationships that could be construed as a potential conflict of interest.

## Publisher's Note

All claims expressed in this article are solely those of the authors and do not necessarily represent those of their affiliated organizations, or those of the publisher, the editors and the reviewers. Any product that may be evaluated in this article, or claim that may be made by its manufacturer, is not guaranteed or endorsed by the publisher.
